# Danger: High Voltage—The Role of Voltage-Gated Calcium Channels in Central Nervous System Pathology

**DOI:** 10.3390/cells6040043

**Published:** 2017-11-15

**Authors:** Andrea Schampel, Stefanie Kuerten

**Affiliations:** 1 Institute of Anatomy and Cell Biology, University of Würzburg, 97070 Würzburg, Germany; andrea.schampel@uni-wuerzburg.de; 2 Institute of Anatomy and Cell Biology, Friedrich-Alexander University Erlangen-Nürnberg, 91054 Erlangen, Germany

**Keywords:** calcium, calcium channel antagonists, CNS, EAE, neurodegeneration, MS, regeneration, remyelination

## Abstract

Voltage-gated calcium channels (VGCCs) are widely distributed within the central nervous system (CNS) and presumed to play an important role in the pathophysiology of a broad spectrum of CNS disorders including Alzheimer’s and Parkinson’s disease as well as multiple sclerosis. Several calcium channel blockers have been in clinical practice for many years so that their toxicity and side effects are well studied. However, these drugs are primarily used for the treatment of cardiovascular diseases and most if not all effects on brain functions are secondary to peripheral effects on blood pressure and circulation. While the use of calcium channel antagonists for the treatment of CNS diseases therefore still heavily depends on the development of novel strategies to specifically target different channels and channel subunits, this review is meant to provide an impulse to further emphasize the importance of future research towards this goal.

## 1. Calcium and Voltage-Gated Calcium Channels (VGCCs)

Calcium is one of the most important intracellular second messengers in the central nervous system (CNS). It regulates numerous cellular processes due to its electrogenic properties. These processes include neurotransmitter release, excitation, cell growth, proliferation, gene expression, long-term potentiation, plasticity and apoptosis [1,2,3]. In order to trigger and maintain Ca^2+^-dependent processes, an influx of cytosolic calcium from the extracytoplasmic space is required. This is achieved by calcium release from internal calcium stores or by entry of calcium ions via the cell membrane. In electrically excitable cells, voltage-gated calcium channels (VGCCs) are the main route for calcium entry into the cell after depolarization of the membrane. Despite mediating calcium influx, VGCCs also regulate intracellular processes depending on their localization. In cardiomyocytes, VGCCs regulate contraction processes; in endocrine cells, they control the secretion of hormones and in the CNS, they modulate the release of neurotransmitters [4]. Structurally, VGCCs are heteromultimeric complexes consisting of a central pore-forming Ca_v_α1 subunit, which is conductive for ions. The central pore-forming subunit is convoyed by several auxiliary subunits (α2δ1-4, β1-4 and γ1-8) [5,6]. So far, ten different Ca_v_α1 subunits have been described and classified according to their pharmacological and electrophysiological properties into high-voltage activated (HVA) and low-voltage activated (LVA) Ca^2+^ channels [4,5]. HVA Ca^2+^ channels include dihydropyridine-sensitive L (“long-lasting”)-type Ca_v_1.1–1.4 and non-L-type Ca_v_2.1–2.3 channels, which are less sensitive for DHP. Compared to LVA channels, which consist of the T (“transient”)-type Ca^2+^ channels Ca_v_3.1–3.3, HVA channels require much stronger depolarization to reach the activation threshold. Additionally, they show prolonged channel opening [4,5,6] (Table 1).

**Figure 1:**
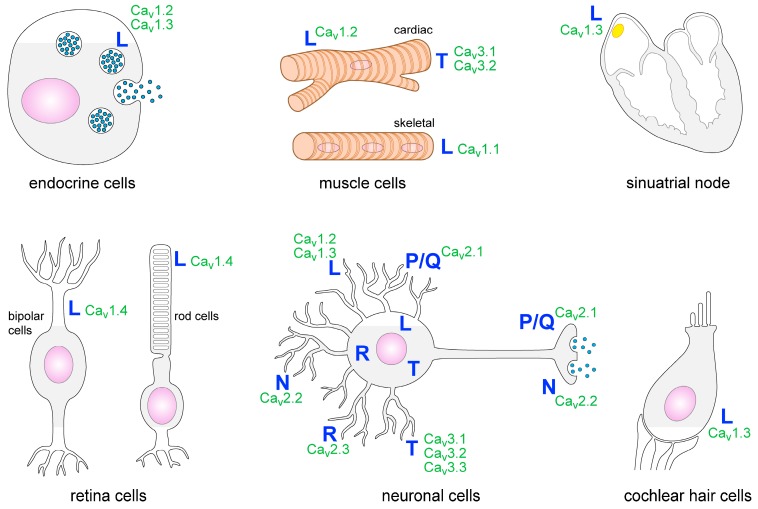
Distribution of VGCC subtypes on different cell types.

Within the nervous system, several types of VGCCs are expressed. They are detectable in many brain areas such as the cortex, thalamus and the hippocampus. P/Q-, T- and N-type VGCCs are the most common ones in the CNS [7]. Presynaptic P/Q- and N-type VGCCs induce neurotransmitter release and T-type VGCCs facilitate rhythmic burst firing of neurons. L-type VGCCs are localized on neuronal cell bodies as well as on dendrites and spines. Postsynaptic L-type VGCCs regulate gene expression and neuronal excitability (Figure 1) [4]. Some types of glial cells such as astrocytes, oligodendrocytes and glial precursor cells have also been shown to express VGCCs [2,8,9,10,11]. Outside the nervous system the heart, skeletal muscle cells, cells of the retina, endocrine cells, cochlear hair cells and cells of the immune system have been reported to express VGCCs or VGCC-like channels (Figure 1) [12,13,14,15].

## 2. Signs of Calcium-Mediated Cellular Damage

Intracytoplasmic calcium levels have to be strictly regulated in order to prevent cellular damage. In the CNS, neuronal organelles such as neurofilaments—and in particular mitochondria—are vulnerable to cytotoxicity [3,16,17,18,19]. Mitochondria play different roles in organisms, which comprise cellular respiration, temporary calcium storage, calcium buffering, maintenance of structural integrity and mediation of apoptosis [19,20,21]. Mitochondrial function can be directly influenced by extracellular signalling molecules. Increased nitric oxide (NO) levels, for instance, can alter gene expression and induce dysfunction of mitochondria. This in turn causes dysregulation of calcium homeostasis, resulting in enhanced cellular degeneration [19] and finally apoptosis of neurons and oligodendrocytes. Mitochondrial dysfunction can be detected ultrastructurally by an increased size (*swelling*) of mitochondria, reflecting an enhanced local energy demand [20,22,23]. Increased intracytoplasmic calcium levels also weaken neuronal integrity as they promote breakdown of the cytoskeleton, including actin, tubulin and intermediate filaments. This becomes evident both histologically and ultrastructurally as cytoplasmic *blebbing* and accumulation or dissolution of filaments [24,25]. Other detectable signs of calcium-mediated damage are dilatation of the endoplasmic reticulum and cytosolic shrinkage [24,25].

## 3. VGCCs in the Pathophysiology and Treatment of CNS Diseases

Studies of human diseases, mouse, rat and cell culture models indicate an important contribution of VGCCs to several neurological and psychiatric disorders, blindness and pain (Table 2) [26]. Of these conditions in particular Parkinson’s and Alzheimer’s disease have been in the focus of research mainly due to their tremendous socioeconomic relevance. In Parkinson’s disease, it has been demonstrated that dihydropyridines—potent VGCC antagonists—reduce the overall population risk in humans [27,28]. Evaluation of the pathogenesis of Alzheimer’s disease has revealed that pathogenic amyloid β (Aβ) peptides elevate L-type VGCC activity in cell cultures [29,30,31,32,33,34,35,36]. In addition, there was increased radiolabel binding to L-type VGCCs in the brains of Alzheimer’s disease patients post mortem [37]. Along these lines, L-type VGCC activity has been reported to be elevated during aging [38,39] and it is assumed to be involved in age-related alterations of synaptic function [38,40], membrane excitability [41] and cognition [42,43]. Yet, there is some controversy because studies of a mouse model of Alzheimer’s disease rather observed a decrease in L-type VGCC currents, suggesting a complex interplay between several factors including aging, the amount of circulating Aβ, Ca^2+^ dysregulation and Ca^2+^ release from the endoplasmic reticulum [44].

The continuous interest in using VGCCs as therapeutic targets to treat CNS disorders is also reflected by currently ongoing clinical trials, of which the majority uses L-type calcium channel antagonists. On the one hand, the L-type calcium channel blocker amlodipine is tested in a trial to reduce the risk for Alzheimer’s disease (NCT02913664) and a phase III trial on the use of the L-type calcium channel antagonist nilvadipine to treat Alzheimer’s disease was recently completed (NCT02017340) with results that are still expected. On the other hand, there are ongoing studies on the efficacy of isradipine in early Parkinson’s disease (NCT02168842). Yet, studies in a mouse model of Parkinson’s disease ask for caution since the plasma concentrations of isradipine approved for therapy were not neuroprotective, most likely due to the fact that the drug fails to reduce somatic calcium oscillations of dopaminergic neurons of the substantia nigra [45]. Isradpine is also currently investigated for cognitive enhancement in schizophrenia and schizoaffective disorder (NCT01658150). In addition, the drug is tested for the treatment of nicotine dependence (NCT03083353). There is one trial using the novel drug CX-8998, a T-type VGCC antagonist for the treatment of essential tremor (NCT03101241). Most recently, nimodipine—a Ca_v_1.2 antagonist—was shown to be neuroprotective in the setting of experimental autoimmune encephalomyelitis—the most common animal model for multiple sclerosis—by limiting microglia-mediated damage of the CNS and promoting remyelination [46]. Interestingly, microglia are devoid of the Ca_v_1.2 channel [46] so that the exact mechanism by which nimodipine acts on microglia still has to be elucidated in future studies. Table 2 summarizes hallmark diseases/syndromes and symptoms that are thought to be associated with VGCC involvement.

Overall, several studies regarding the role of VGCCs in CNS pathology exist and several attempts have been made to use VGCC antagonists as therapeutic targets in this context. While historically, VGCCs were targets of the first synthesized drugs [6], the establishment of ion channel-specific therapies for CNS disorders has so far proven to be difficult. Yet, the availability of a broad range of modern technologies such as RNAi, function-blocking antibodies and gene-editing present promising therapeutic avenues, which may be of particular importance for several still incurable and devastating CNS disorders including Alzheimer’s’ and Parkinson’s disease as well as multiple sclerosis.

## Figures and Tables

**Table 1:** Classification of voltage-gated calcium channels (VGCCs) according to their voltage-dependent activation.

**High-Voltage Activated**	**Family**
L-type (“long-lasting”) VGCC	Ca_v_1.1–Ca_v_1.4
P-type (“Purkinje cell”)/Q-type VGCC	Ca_v_2.1
N-type (“neural”) VGCC	Ca_v_2.2
R-type (“residual”/“resistant”) VGCC	Ca_v_2.3
**Low-Voltage Activated**	**Family**
T-type (“transient”) VGCC	Ca_v_3.1–Ca_v_3.3

**Table 2:** Involvement of VGCCs in neurologic and psychiatric disorders.

Channel	Disease/Symptom	Species
Ca_v_1.2	Autism/Timothy syndrome	Human [47]
	Conditioned fear	Mouse [48]
	Depression/Mood disorders	Human, mouse [49,50]
	Febrile seizures	Rat [51]
	Multiple sclerosis	Mouse [46]
	Pain	Mouse, rat [52,53,54]
	Parkinson’s disease	Human, mouse (reviewed in [28])
	Schizophrenia	Human [55]
Ca_v_1.3	Deafness	Mouse [56,57]
	Depression	Human, mouse [50,58]
	Pain	Rat [52,53]
	Parkinson’s disease	Human, mouse (reviewed in [28])
Ca_v_1.4	(Incomplete X-linked congenital stationary) night blindness	Human [59,60]
Ca_v_2.1	Episodic ataxia type 2 and familiar hemiplegic migraine type 1	Human [61,62,63]
	Spinocerebellar ataxia 6	Human [64,65]
Ca_v_2.2	Pain	Mouse [54]
Ca_v_2.3	Anxiety	Mouse [66]
	Absence epilepsy	Mouse [66]
	Pain	Mouse [67]
Ca_v_3.1	Thalamocortical network activity/ absence epilepsy	Mouse [68]
Ca_v_3.1–3.3	Autism/Autism spectrum disorders	Human [69]
	Pain	Human, mouse, rat (reviewed in [70])
	Parkinson’s disease/locomotor deficits	Rat [71]
Various VGCCs	Alzheimer’s disease/dementia	Mouse, rat, human (reviewed in [72])

## Abbreviations

Aβ	Amyloid β
CNS	Central nervous system
HVA	High-voltage activated
LVA	Low-voltage activated
VGCC	Voltage-gated calcium channel

## References

[1] Yagami T., Kohma H., Yagamoto Y. (2012). L-type voltage-dependent calcium channels as therapeutic targets for neurodegenerative diseases. Curr. Med. Chem..

[2] Braet K., Cabooter L., Paemeleire K., Leybaert L. (2004). Calcium signal communication in the central nervous system. Biol. Cell.

[3] Young W. (1992). Role of calcium in central nervous system injuries. J. Neurotrauma.

[4] Catterall W.A., Perez-Reyes E., Snutch T.P., Striessnig J. (2005). International Union of Pharmacology. XLVIII. Nomenclature and structure-function relationships of voltage-gated calcium channels. Pharmacol. Rev..

[5] Wormuth C., Lundt A., Henseler C., Müller R., Broich K., Papazoglou A., Weiergräber M. (2016). Ca_v_2.3 R-type voltage-gated Ca^2+^ channels—Functional implications in convulsive and non-convulsive seizure activity. Open Neurol. J..

[6] Waszkielewicz A.M., Gunia A., Szkaradek N., Słoczyńska K., Krupińska S., Marona H. (2013). Ion channels as drug targets in central nervous system disorders. Curr. Med. Chem..

[7] Schlick B., Flucher B.E., Obermair G.J. (2010). Voltage-activated calcium channel expression profiles in mouse brain and cultured hippocampal neurons. Neuroscience.

[8] Verkhratsky A., Kettenmann H. (1996). Calcium signalling in glial cells. Trends Neurosci..

[9] Casamassima F., Hay A.C., Benedetti A., Lattanzi L., Cassano G.B., Perlis R.H. (2010). L-type calcium channels and psychiatric disorders: A brief review. Am. J. Med. Genet. B Neuropsychiatr. Genet..

[10] Triggle D.J. (2007). Calcium channel antagonists: Clinical uses—Past, present and future. Biochem. Pharmacol..

[11] Silei V., Fabrizi C., Venturini G., Salmona M., Bugiani O., Tagliavini F., Lauro G.M. (1999). Activation of microglial cells by PrP and beta-amyloid fragments raises intracellular calcium through L-type voltage sensitive calcium channels. Brain Res..

[12] Seitter H., Koschak A. (2017). Relevance of tissue specific subunit expression in channelopathies. Neuropharmacology.

[13] Lyubchenko T. (2010). Ca^2+^ signalling in B cells. Sci. World J..

[14] Sadighi Akha A.A., Willmott N.J., Brickley K., Dolphin A.C., Galione A., Hunt S.V. (1996). Anti-Ig-induced calcium influx in rat B lymphocytes mediated by cGMP through a dihydropyridine-sensitive channel. J. Biol. Chem..

[15] Mesirca P., Torrente A.G., Mangoni M.E. (2015). Functional role of voltage gated Ca(^2+^) channels in heart automaticity. Front. Physiol..

[16] Stys P.K., Jiang Q. (2002). Calpain-dependent neurofilament breakdown in anoxic and ischemic rat central axons. Neurosci. Lett..

[17] Takano Y., Ohguro H., Dezawa M., Ishikawa H., Ohguro I., Mamiya K., Metoki T., Ishikawa F., Nakazawa M. (2004). Study of drug effects of calcium channel blockers on retinal degeneration of rd mouse. Biochem. Biophys. Res. Commun..

[18] Damaj M.I., Martin B.R. (1993). Calcium agonists and antagonists of the dihydropyridine type: Effect on nicotine-induced antinociception and hypomotility. Drug Alcohol Depend..

[19] Gonsette R.E. (2008). Oxidative stress and excitotoxicity: A therapeutic issue in multiple sclerosis?. Mult. Scler. J..

[20] Waxman S.G. (2006). Axonal conduction and injury in multiple sclerosis: The role of sodium channels. Nat. Rev. Neurosci..

[21] Zambonin J.L., Zhao C., Ohno N., Campbell G.R., Engeham S., Ziabreva I., Schwarz N., Lee S.E., Frischer J.M., Turnbull D.M. (2011). Increased mitochondrial content in remyelinated axons: Implications for multiple sclerosis. Brain.

[22] Dhib-Jalbut S., Arnold D.L., Cleveland D.W., Fisher M., Friedlander R.M., Mouradian M.M., Przedborski S., Trapp B.D., Wyss-Coray T., Yong V.W. (2006). Neurodegeneration and neuroprotection in multiple sclerosis and other neurodegenerative diseases. J. Neuroimmunol..

[23] Dutta R., McDonough J., Yin X., Peterson J., Chang A., Torres T., Gudz T., Macklin W.B., Lewis D.A., Fox R.J. (2006). Mitochondrial dysfunction as a cause of axonal degeneration in multiple sclerosis patients. Ann. Neurol..

[24] Trump B.F., Berezesky I.K. (1995). Calcium-mediated cell injury and cell death. FASEB J..

[25] Soellner I.A., Rabe J., Mauri V., Kaufmann J., Addicks K., Kuerten S. (2013). Differential aspects of immune cell infiltration and neurodegeneration in acute and relapse experimental autoimmune encephalomyelitis. Clin. Immunol..

[26] Heyes S., Pratt W.S., Rees E., Dahimene S., Ferron L., Owen M.J., Dolphin A.C. (2015). Genetic disruption of voltage-gated calcium channels in psychiatric and neurological disorders. Prog. Neurobiol..

[27] Ritz B., Rhodes S.L., Qian L., Schernhammer E., Olsen J.H., Friis S. (2010). L-type calcium channel blockers and Parkinson disease in Denmark. Ann. Neurol..

[28] Surmeier D.J., Schumacker P.T., Guzman J.D., Ilijic E., Yang B., Zampese E. (2017). Calcium and Parkinson’s disease. Biochem. Biophys. Res. Commun..

[29] Davidson R.M., Shajenko L., Donta T.S. (1994). Amyloid beta-peptide (A beta P) potentiates a nimodipine-sensitive L-type barium conductance in N1E-115 neuroblastoma cells. Brain Res..

[30] Fu H., Li W., Lao Y., Luo J., Lee N.T., Kan K.K., Tsang H.W., Tsim K.W., Pang Y., Li Z. (2006). Bis(7)-tacrine attenuates beta amyloid-induced neuronal apoptosis by regulating L-type calcium channels. J. Neurochem..

[31] Kim S., Rhim H. (2011). Effects of amyloid-beta peptides on voltage-gated L-type Ca(V)1.2 and Ca(V)1.3 Ca (^2+^) channels. Mol. Cells.

[32] Ramsden M., Henderson Z., Pearson H.A. (2002). Modulation of Ca^2+^ channel currents in primary cultures of rat cortical neurones by amyloid beta protein (1–40) is dependent on solubility status. Brain Res..

[33] Scragg J.L., Fearon I.M., Boyle J.P., Ball S.G., Varadi G., Peers C. (2005). Alzheimer’s amyloid peptides mediate hypoxic up-regulation of L-type Ca^2+^ channels. FASEB J..

[34] Ueda K., Shinohara S., Yagami T., Asakura K., Kawasaki K. (1997). Amyloid beta protein potentiates Ca^2+^ influx through L-type voltage-sensitive Ca^2+^ channels: A possible involvement of free radicals. J. Neurochem..

[35] Webster N.J., Ramsden M., Boyle J.P., Pearson H.A., Peers C. (2006). Amyloid peptides mediate hypoxic increase of L-type Ca^2+^ channels in central neurons. Neurobiol. Aging.

[36] Weiss J.H., Pike C.J., Cotman C.W. (1994). Ca^2+^ channel blockers attenuate beta-amyloid peptide toxicity to cortical neurons in culture. J. Neurochem..

[37] Coon A.L., Wallace D.R., Mactutus C.F., Booze R.M. (1999). L-type calcium channels in the hippocampus and cerebellum of Alzheimer’s disease brain tissue. Neurobiol. Aging.

[38] Thibault O., Landfield P.W. (1996). Increase in single L-type calcium channels in hippocampal neurons during aging. Science.

[39] Norris C.M., Blalock E., Chen K.C., Porter N.M., Thibault O., Kraner S.D., Landfield P.W. (2010). Hippocampal ‘zipper’ slice studies reveal a necessary role for calcineurin in the increased activity of L-type Ca(^2+^) channels with aging. Neurobiol. Aging.

[40] Norris C.M., Halpain S., Foster T.C. (1998). Reversal of age-related alterations in synaptic plasticity by blockade of L-type Ca^2+^ channels. J. Neurosci..

[41] Moyer J.R., Thompson L.T., Black J.P., Disterhoft J.F. (1992). Nimodipine increases excitability of rabbit CA1 pyramidal neurons in an age- and concentration-dependent manner. J. Neurophysiol..

[42] Deyo R.A., Straube K.T., Disterhoft J.F. (1989). Nimodipine facilitates associative learning in aging rabbits. Science.

[43] Veng L.M., Mesches M.H., Browning M.D. (2003). Age-related working memory impairment is correlated with increases in the L-type calcium channel protein alpha1D (Cav1.3) in area CA1 of the hippocampus and both are ameliorated by chronic nimodipine treatment. Brain Res. Mol. Brain Res..

[44] Thibault O., Pancani T., Landfield P.W., Norris C.M. (2012). Reduction in neuronal L-type calcium channel activity in a double knock-in mouse model of Alzheimer’s disease. Biochim. Biophys. Acta.

[45] Ortner N.J., Bock G., Dougalis A., Kharitonova M., Duda J., Hess S., Tuluc P., Pomberger T., Stefanova N., Pitterl F. (2017). Lower affinity of isradipine for L-Type Ca^2+^ channels during substantia nigra dopamine neuron-like activity: Implications for neuroprotection in Parkinson's disease. J. Neurosci..

[46] Schampel A., Volovitch O., Koeniger T., Scholz C.J., Jörg S., Linker R.A., Wischmeyer E., Wunsch M., Hell J.W., Ergün S. (2017). Nimodipine fosters remyelination in a mouse model of multiple sclerosis and induces microglia-specific apoptosis. Proc. Natl. Acad. Sci. USA.

[47] Splawski I., Timothy K.W., Decher N., Kumar P., Sachse F.B., Beggs A.H., Sanguinetti M.C., Keating M.T. (2005). Severe arrhythmia disorder caused by cardiac L-type calcium channel mutations. Proc. Natl. Acad. Sci. USA.

[48] Busquet P., Hetzenauer A., Sinnegger-Brauns M.J., Striessnig J., Singewald N. (2008). Role of L-type Ca^2+^ channel isoforms in the extinction of conditioned fear. Learn. Mem..

[49] Dao D.T., Mahon P.B., Cai X., Kovacsics C.E., Blackwell R.A., Arad M., Shi J., Zandi P.P., O’Donnell P., Bipolar Genome Study (BiGS) Consortium (2010). Mood disorder susceptibility gene CACNA1C modifies mood-related behaviors in mice and interacts with sex to influence behavior in mice and diagnosis in humans. Biol. Psychiatry.

[50] Ostacher M.J., Iosifescu D.V., Hay A., Blumenthal S.R., Sklar P., Perlis R.H. (2014). Pilot investigation of isradipine in the treatment of bipolar depression motivated by genome-wide association. Bipolar Disord..

[51] Radzicki D., Yau H.J., Pollema-Mays S.L., Mlsna L., Cho K., Koh S., Martina M. (2013). Temperature-sensitive Cav1.2 calcium channels support intrinsic firing of pyramidal neurons and provide a target for the treatment of febrile seizures. J. Neurosci..

[52] Roca-Lapirot O., Radwani H., Aby F., Nagy F., Landry M., Fossat P. (2017). Calcium signalling through L-type calcium channels: Role in pathophysiology of spinal nociceptive transmission. Br. J. Pharmacol..

[53] Radwani H., Lopez-Gonzalez M.J., Cattaert D., Roca-Lapirot O., Dobremez E., Bouali-Benazzouz R., Eiríksdóttir E., Langel Ü., Favereaux A., Errami M. (2016). Ca_v_1.2 and Ca_v_1.3 L-type calcium channels independently control short- and long-term sensitization to pain. J. Physiol..

[54] Gadotti V.M., Bladen C., Zhang F.X., Chen L., Gündüz M.G., Simsek R., Safak C., Zamponi G.W. (2015). Analgesic effect of a broad-spectrum dihydropyridine inhibitor of voltage-gated calcium channels. Pflugers Arch..

[55] Ripke S., O’Dushlaine C., Chambert K., Moran J.L., Kahler A.K., Akterin S., Bergen S.E., Collins A.L., Crowley J.J., Fromer M. (2013). Genome-wide association analysis identifies 13 new risk loci for schizophrenia. Nat. Genet..

[56] Nouvian R., Beutner D., Parsons T.D., Moser T. (2006). Structure and function of the hair cell ribbon synapse. J. Membr. Biol..

[57] Platzer J., Engel J., Schrott-Fischer A., Stephan K., Bova S., Chen H., Zheng H., Striessnig J. (2000). Congenital deafness and sinoatrial node dysfunction in mice lacking class D L-type Ca^2+^ channels. Cell.

[58] Orthner N.J., Striessnig J. (2016). L-type calcium channels as drug targets in CNS disorders. Channels.

[59] Striessnig J., Hoda J.C., Koschak A., Zaghetto F., Müllner C., Sinnegger-Brauns M.J., Wild C., Watschinger K., Trockenbacher A., Pelster G. (2004). L-type Ca^2+^ channels in Ca^2+^ channelopathies. Biochem. Biophys. Res. Commun..

[60] Bech-Hansen N.T., Naylor M.J., Maybaum T.A., Pearce W.G., Koop B., Fishman G.A., Mets M., Musarella M.A., Boycott K.M. (1998). Loss-of-function mutations in a calcium-channel α1 subunit gene in Xp11.23 cause incomplete X-linked congenital stationary night blindness. Nat. Genet..

[61] Nachbauer W., Nocker M., Karner E., Stankovic I., Unterberger I., Eigentler A., Schneider R., Poewe W., Delazer M., Boesch S. (2014). Episodic ataxia type 2: Phenotype characteristics of a novel CACNA1A mutation and review of the literature. J. Neurol..

[62] Baloh R.W., Jen J.C. (2002). Genetics of familial episodic vertigo and ataxia. Ann. N. Y. Acad. Sci..

[63] Van den Maagdenberg A.M., Pietrobon D., Pizzorusso T., Kaja S., Broos L.A., Cesetti T., van den Ven R.C., Tottene A., van der Kaa J., Plomp J.J. (2004). A Cacna1a knockin migraine mouse model with increased susceptibility to cortical spreading depression. Neuron.

[64] Matsuyama Z., Kawakami H., Maruyama H., Izumi Y., Komure O., Udaka F., Kameyama M., Nishio T., Kuroda Y., Nishimura M. (1997). Molecular features of the CAG repeats of spinocerebellar ataxia 6 (SCA6). Hum. Mol. Genet..

[65] Zhuchenko O., Bailey J., Bonnen P., Ashizawa T., Stockton D.W., Amos C., Dobyns W.B., Subramony S.H., Zoghbi H.Y., Lee C.C. (1997). Autosomal dominant cerebellar ataxia (SCA6) associated with small polyglutamine expansions in the alpha 1A-voltage-dependent calcium channel. Nat. Genet..

[66] Zaman T., Lee K., Park C., Paydar A., Choi J.H., Cheong E., Lee C.J., Shin H.S. (2011). Ca_v_2.3 channels are critical for oscillatory burst discharges in the reticular thalamus and absence epilepsy. Neuron.

[67] Saegusa H., Kurihara T., Zong S., Minowa O., Kazuno A., Han W., Matsuda Y., Yamanaka H., Osanai M., Noda T. (2000). Altered pain responses in mice lacking α1E subunit of the voltage-dependent Ca^2+^ channel. Proc. Natl. Acad. Sci. USA.

[68] Ernst W.L., Zhang Y., Yoo J.W., Ernst S.J., Noebels J.L. (2009). Genetic enhancement of thalamocortical network activity by elevating α1G-mediated low-voltage-activated calcium current induces pure absence epilepsy. J. Neurosci..

[69] Lu A.T., Dai X., Martinez-Agosto J.A., Cantor R.M. (2012). Support for calcium channel gene defects in autism spectrum disorders. Mol. Autism.

[70] Bourinet E., Francois A., Laffray S. (2016). T-type channels in neuropathic pain. Pain.

[71] Tai C.H., Yang Y.C., Pan M.K., Huang C.S., Kuo C.C. (2011). Modulation of subthalamic T-type Ca^2+^ channels remedies locomotor deficits in a rat model of Parkinson disease. J. Clin. Investig..

[72] Nimrich V., Eckert A. (2013). Calcium channel blockers and dementia. Br. J. Pharmacol..

